# Emerging Spectrum of Perforation Peritonitis in Developing World

**DOI:** 10.3389/fsurg.2020.00050

**Published:** 2020-09-15

**Authors:** Tariq Hameed, Awadh Kumar, Shivanand Sahni, Rahul Bhatia, Ajit Kumar Vidhyarthy

**Affiliations:** ^1^Department of Surgery, Hamdard Institute of Medical Sciences & Research, New Delhi, India; ^2^Department of Surgery, Darbhanga Medical College and Hospital, Darbhanga, India; ^3^Department of Surgery, Maulana Azad Medical College, New Delhi, India

**Keywords:** Gastrointestinal tract, perforation, peritonitis, etiology, pneumoperitoneum, exploratory laparotomy, seasonal variation

## Abstract

**Background:** Gastrointestinal perforations constitute a major cause of patients with acute abdomen pain coming to the surgery emergency room. Incidence, site of perforation, and age is different in the developing world and is showing new trends. The etiological spectrum in the developing world is different from the western world. This study was conducted to find out the latest trends in perforation peritonitis in India.

**Methods:** This study was conducted in a single surgical unit of Darbhanga Medical College and Hospital, India. A total of 350 consecutive patients with perforation peritonitis were studied in terms of age, sex, seasonal variation, biochemical parameters, clinical presentation, radiological and intraoperative findings, surgical intervention, and postoperative outcome.

**Results:** The most common cause of perforation peritonitis in our study was a duodenal ulcer (~50%) followed by typhoid (20%), traumatic (14.5%), appendicular (7.4%), and tubercular (3.1%) cases. Males were three times more commonly affected than females. Peak incidence was noted in the 2^nd^ and 3^rd^ decades of life. Peptic ulcer perforations were common in autumn and winter and typhoid perforations were common during the summer and rainy seasons.

**Conclusion:** Spectrum of perforation peritonitis cases in this part of world is different from developed western countries. It is different in respect of younger age at presentation, site of perforation, and etiological factors. Infective pathology makes up to a quarter of total cases in the developing world. The developing world has more perforation peritonitis cases involving the upper gastrointestinal tract, while the western world has a predominance of lower gastrointestinal tract perforations.

## Background

Gastrointestinal perforations as a sequelae to various disease processes, trauma, and diagnostic/therapeutic procedures constitute a major percentage of acute abdominal emergencies (1, 2). Gastrointestinal perforations lead to diffuse peritonitis, toxemia, septicemia, metabolic and circulatory instability, renal failure, and pulmonary insufficiency, compounded by advanced age and delay in therapeutic procedures, it leads to high mortality and morbidity (3). Various studies have shown different etiological spectrums for perforation peritonitis in India compared to rest of the world (4–6). Our study was undertaken to find the emerging spectrum of perforation peritonitis in patients requiring emergency surgery in Darbhanga Medical College and Hospital, the biggest tertiary care facility in Bihar, India catering to patients mostly from rural areas.

## Materials and Methods

This prospective study was conducted at a single surgical unit in the Department of Surgery, Darbhanga Medical College and Hospital, Darbhanga, India from October 2012 to June 2018. The study population included 350 patients who presented a surgical emergency of perforation peritonitis at Darbhanga Medical College and Hospital, who underwent an exploratory laparotomy.

The diagnosis of gastrointestinal perforation was made on the basis of detailed history, physical examination, radiological investigations, and operative findings. Associated comorbidity conditions and postoperative courses were noted for each patient. Exploratory laparotomy patients were managed according to the site of perforation and managed in a postoperative ward. All patients were placed on parenteral nutrition and broad spectrum antibiotics, oral feeding was resumed once bowel sounds were present. Peritoneal contamination was treated by intraoperative peritoneal lavage with 2% povidone iodine, normal saline, and metronidazole solution. In all the cases of gastrointestinal perforations broad spectrum antibiotics were started pre-operatively. Postoperatively, patients were managed by intravenous fluid and electrolytes, antibiotics, analgesics, nasogastric aspirations, and chest physiotherapy. Normal bowel sound returned, on average, within 3.3 days. Nasogastric tubes were removed after an average of 4.2 days and oral fluid was started, on average, after 4.8 days.

### Inclusion Criteria

All patients with cases of peritonitis caused by gastrointestinal tract perforations who were undergoing exploratory laparotomies were included in the study.

### Exclusion Criteria

Cases of primary peritonitis, iatrogenic perforations, and anastomosis leak were excluded from the study. Perforation peritonitis cases due to corrosive ingestion were also excluded.

## Results

**Figure 1:**
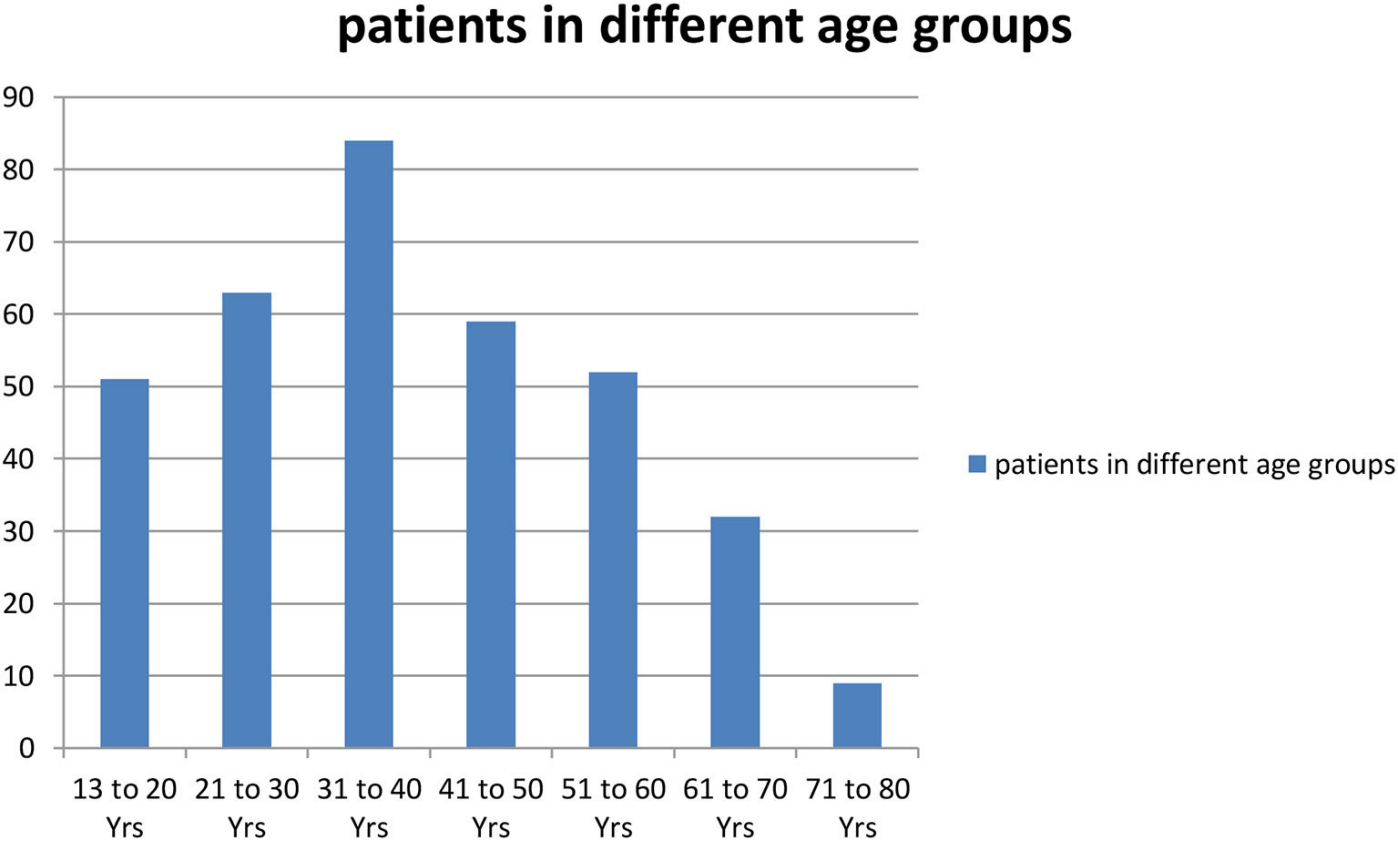
Number of patients in different age groups.

**Figure 2:**
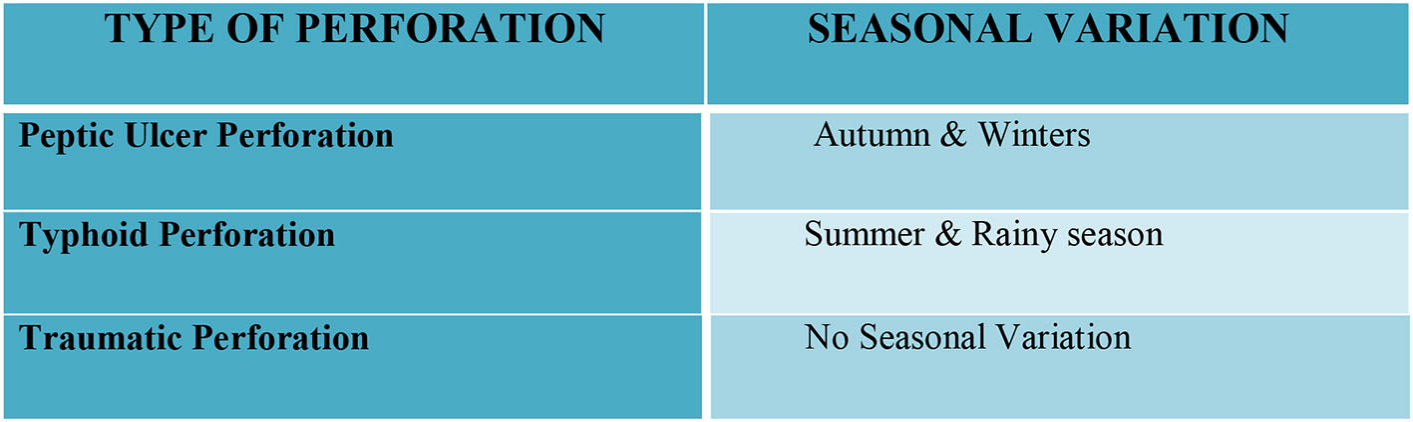
Seasonal predominance of different perforations.

In total, 350 consecutive patients with perforation peritonitis were studied. The mean age of presentation was 39.6 years and ranged from 13 to 78 years. Peak incidence was noted in the 2^nd^ and 3^rd^ decades of life (Figure 1). The majority of patients in our study were male, 268 (76.6%) while there were 82 (23.4%) female patients. Male patients were common in all types of perforation peritonitis cases, overall male to female ratio was 3.3:1. Peak incidence of peptic ulcer perforation was seen during the autumn and winter seasons. Typhoid perforation was common during the rainy and summer seasons, traumatic perforation showed no seasonal variation (Figure 2). The common presenting symptoms in gastrointestinal perforations were pain, distension, and constipation followed by vomiting, fever, diarrhea, and melena. Signs of dehydration, shock, and anemia were present in 42.2%, 28%, and 59.4% of patients, respectively. Preoperative plain x-rays of the abdomen in erect or sitting posture showed pneumoperitoneum in 80% of cases, features of peritonitis in 39.2% cases, and features of acute intestinal obstruction (multiple airfluid levels) in only 4% of cases. Serum sodium was found to be <126.0 meEq/liter in 14.6% cases and serum potassium was <3.6 meEq/liter in 20.9% cases.

**Figure 3:**
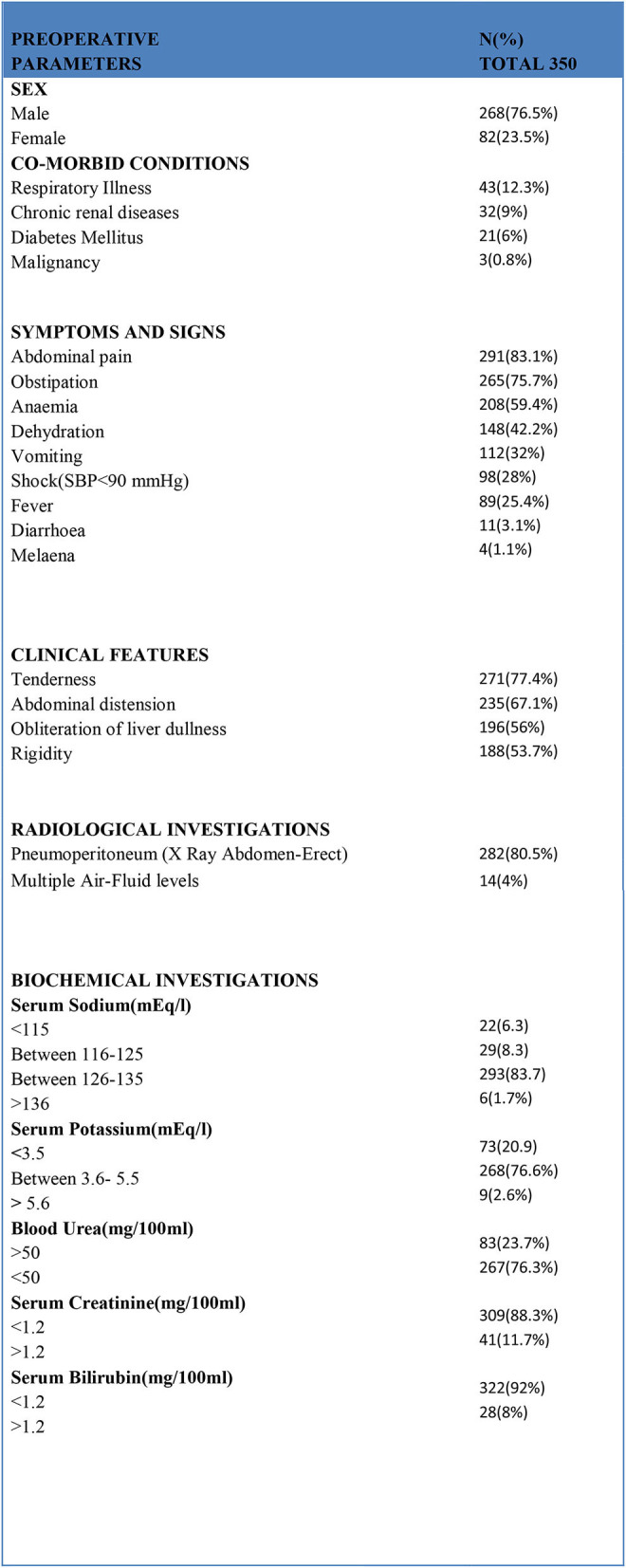
Various preoperative parameters.

Preoperative blood urea (>50 mg/100 ml) and serum creatinine (>1.2/100 ml) values were raised in 83 and 41 patients, respectively. Serum bilirubin was more than 1.2 mg/100 ml in 28 patients (Figure 3).

In our study the most common cause of gastrointestinal perforation was peptic ulcers (182 cases) followed by typhoid (70 cases), trauma (51 cases), acute appendicitis (26 cases), and tuberculosis (11 cases), in that order. Other causes were Meckel's diverticulitis, non-specific perforation, perforation with malignancy, and cecal amoebic perforation.

**Figure 4:**
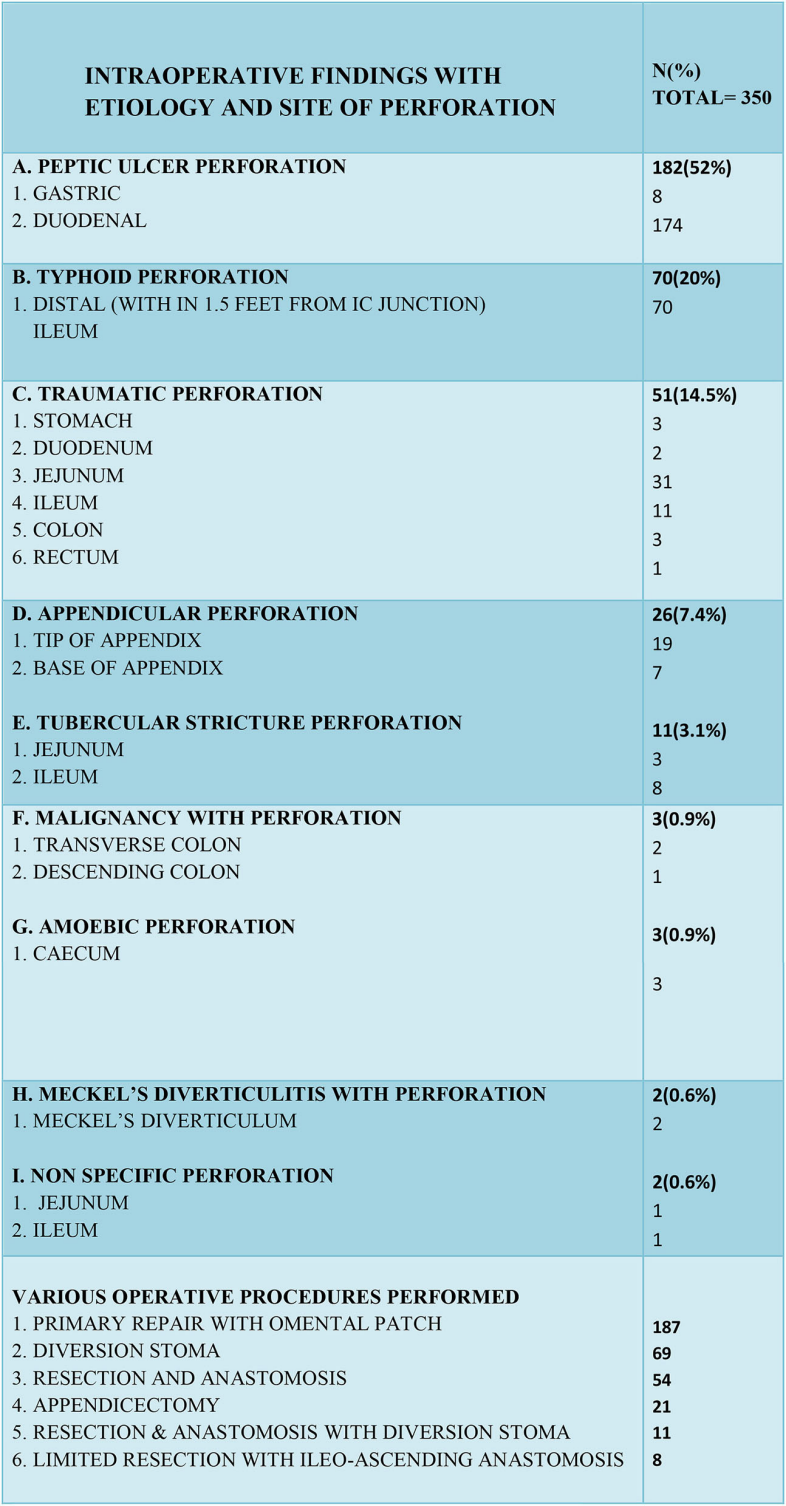
Intraoperative findings with operative management.

In peptic ulcer perforation, the most common site of perforation was first part of the duodenum. The site of typhoid perforation was the distal ileum, within two feet of the ileocecal junction. The sites of traumatic perforation were stomach, jejunum, ileum, colon, duodenum, and rectum, jejunum being the most common of all (31 cases). 73.1% of appendicular perforations occurred at the tip of the appendix and the rest at the base of appendix. The most common site of tubercular perforation was the terminal ileum (8 cases) and then the jejunum (3 cases) (Figure 4).

In peptic ulcer perforation, primary closure with an omental patch repair was completed. In most enteric perforation cases with fecal peritonitis or with septic shock, an ileostomy was performed, primary repair with resection and anastomosis was performed in younger patients (<40 years) who had minimal fecal contamination and were hemodynamically stable with their biochemical parameters in a normal range. In traumatic perforations of <2 cm in size, primary closure of perforation was performed and in larger or multiple perforations in a segment or in cases where a mesenteric tear was causing gangrene of a segment of bowel, it was treated with resection and end to end anastomosis. For colonic and rectal perforations, a diversion stoma was made after performing primary repair. Appendicular perforations were treated with appendicectomy and in five cases limited resection with ileo-ascending anastomosis was done. Tubercular stricture perforations were treated mainly with resection-anastomosis and in few cases a diversion stoma was created. In amoebic cecal perforations, limited resection with ileoascending anastomosis was performed (Figure 4).

**Figure 5:**
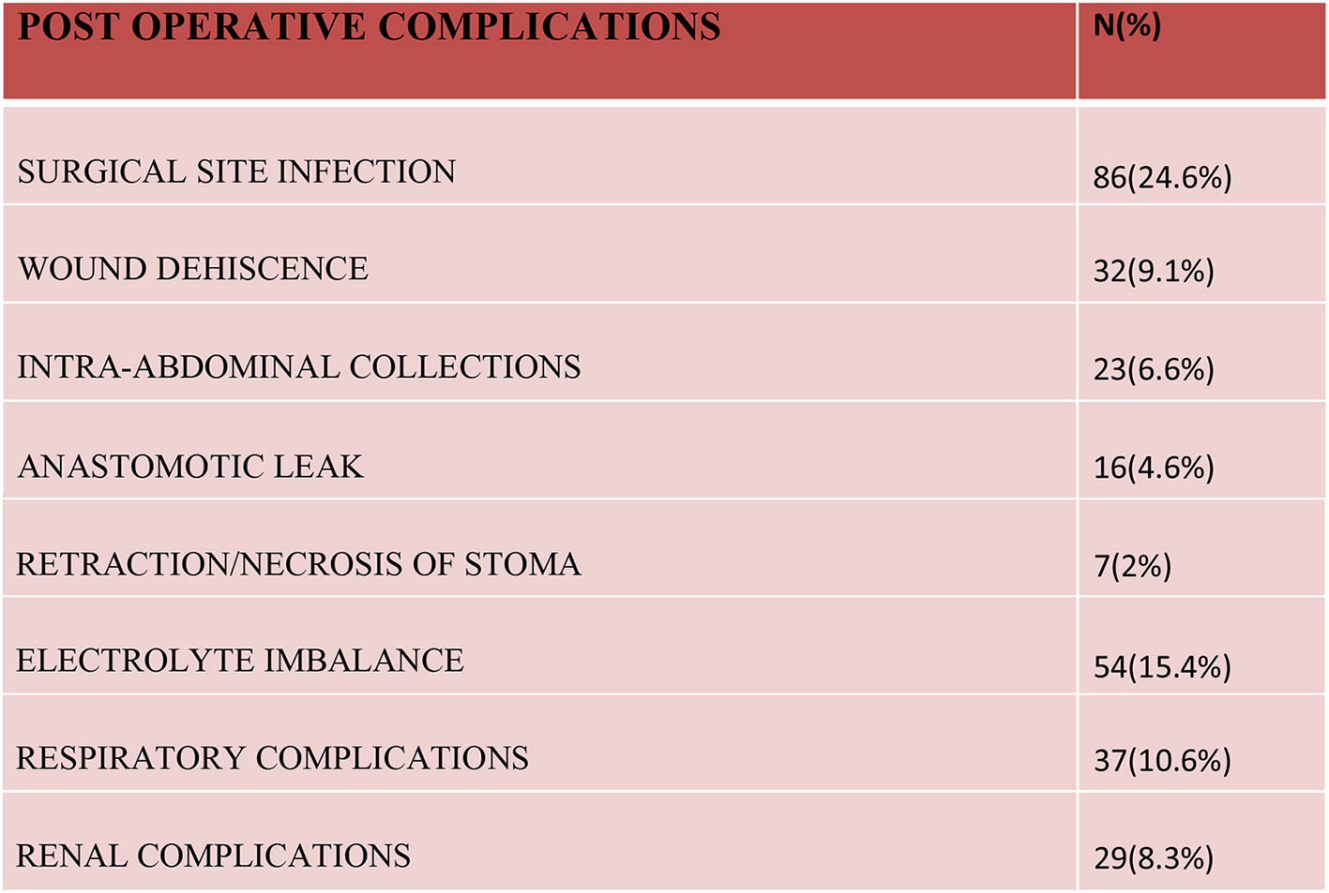
Postoperative complications.

A surgical site infection (~24.6%), both superficial and deep, was the most common complication noted in our study. A burst abdomen was noticed in more than 9% of cases and intra-abdominal pus collection was seen in 6.6% of cases. Delayed secondary suturing was performed for burst abdomens and intra-abdominal collections were managed by ultrasound-guided drain/aspiration in almost all the cases. Sixteen cases had anastomosis site leaks, which required re-exploration in all cases. In the postoperative period, 54 patients developed an electrolyte imbalance in the form of hypokalemia and hyponatremia, it was most common on the 1^st^ and 2^nd^ postoperative days. Hypokalemia was noted in 37 patients and hyponatremia was present in 23 patients. Respiratory issues and renal insufficiency was observed in patients who had pre-existing symptoms of such illnesses and many of them were already under treatment for the same (Figure 5).

## Discussion

Perforation peritonitis remains the most common surgical emergency encountered in a tropical country like India and age distribution is also relatively lower than seen in the western world (6–9). In our study, the time between the onset of symptoms to presentation in hospital was longer (>48 h) and was associated with more fecal contamination and features of frank peritonitis in the majority of cases. Peritonitis due to proximal gastrointestinal perforations were more common in the developing world than distal gastrointestinal perforations which are more common in the western world (10–12). Gastroduodenal perforations cases, a total of 52%, were found to be the most common cause of perforation peritonitis and it was almost the same as found in many other recent studies in India and Pakistan but was different to the western world where more than 48% of cases were due to penetrating trauma and 21% of cases were due to appendicular perforation (13–16). Sartelli et al. in their series reported 158 colonic non-diverticular perforations, 103 small bowel perforations, and 100 appendicular perforations with generalized peritonitis but the number of gastroduodenal perforations was only 156, highlighting the difference of etiology from our series (8). A multicenter French study also showed that ~63% of peritonitis cases were because of appendicular and colonic perforations and only 18% were because of gastroduodenal perforations (12). In a retrospective observational study conducted by Ross et al. the proportion of colonic and appendicular perforations was far higher than gastroduodenal perforations (9). Infectious pathology namely typhoid, tuberculosis, and amoebic perforations remain an important etiological factor of perforation peritonitis in the eastern part of the world and accounted for almost a quarter (24%) of all cases in our study, it is in sharp contrast to the western world where only 2.7% of the cases were due to infectious pathology (14). A study from Pakistan had ~43% cases due to tuberculosis and typhoid, it highlights the role of infectious pathology as a leading cause of perforation peritonitis in the developing world (16). In the present series, traumatic perforation was found to be the cause in 14.4% of cases, which was higher than previously reported in studies by Jhobta et al. (9%) and Chakma et al. (8.57%) (15, 17). Traumatic perforation was the third most common cause of perforation peritonitis in this series ahead of appendicular and tubercular stricture perforation. This change in spectrum is most likely because of the development of good highways associated with more road traffic accidents in rural areas. Another interesting trend noticed in our study is declining share of tubercular perforations (3.1%) as compared to two other studies in the recent past, it is because of early diagnosis of tuberculosis and better treatment facilities available at a primary healthcare level along with increased awareness (13, 16). The share of appendicular perforation in our study is considerably lower at 7.4% than previously reported in other studies (13, 15, 17), it might be because of early intervention and availability of surgical facilities in rural areas too. Afridi et al. had a 5% incidence rate of appendicular perforation peritonitis, which is in tandem with our finding. In our study <1% of cases were due to malignancy, which is in stark contrast to the developed world where malignancy accounts for ~15–20% of cases of perforation peritonitis (9, 18, 19). Seasonal variation was also noted in our study. Peak incidence of peptic ulcer perforation was seen during autumn and winter seasons. Enteric perforation was common during rainy and summer seasons. A study by Agbonrofo et al. showed two peaks of seasonal variation for gastroduodenal perforations, one in February to May (40%) and a second in August to October (32%), Kemparaj et al. showed higher number of perforations (39%) in the July to October period (20, 21). In our study mortality rate was found to be 6% which is in accordance with many other studies showing mortality rate in the same range (8, 22). Mortality is associated with delayed presentation to hospital, old age, and co-morbid conditions like respiratory illnesses, chronic renal disease, and diabetes mellitus. Proper resuscitation before exploration leads to better postoperative results by decreasing morbidity and mortality rates.

## Conclusion

The spectrum of perforation peritonitis cases in this part of world is different from developed western countries. It is different in respect of younger age, site of perforation, and etiological factors. Duodenal perforation remains the single biggest cause of perforation peritonitis accounting for almost half of the total cases. Infective pathology remains a major entity causing a quarter of all perforation peritonitis cases. Another important finding noted in our study is the emergence of trauma as an important cause of perforation peritonitis, it is the third most common cause of perforation peritonitis in our study. One noticeable finding is the declining share of tubercular perforation in perforation peritonitis cases. The outcome depends on many factors; pre-operative rehydration, correction of electrolyte imbalances, and taking care of comorbid conditions. Early diagnosis and fast surgical intervention is associated with a good outcome.

## Data Availability Statement

All datasets generated for this study are included in the article/supplementary material.

## Ethics Statement

The studies involving human participants were reviewed and approved by Darbhanga Medical College, Darbhanga, Bihar, India. The patients/participants provided their written informed consent to participate in this study.

## Author Contributions

TH, AK, and SS conceived the research topic and designed this study. TH and AV were actively involved in acquisition of data analysis and interpretation of data was done by RB, TH, AK, and SS. Drafting of manuscript was done by TH, AK, and RB. Manuscript was critically revised by SS and AV. Final version of manuscript was approved by all authors.

## Conflict of Interest

The authors declare that the research was conducted in the absence of any commercial or financial relationships that could be construed as a potential conflict of interest.
